# Long-Term Efficacy and Safety of Acoramidis in ATTR-CM: Initial Report From the Open-Label Extension of the ATTRibute-CM Trial

**DOI:** 10.1161/CIRCULATIONAHA.124.072771

**Published:** 2024-11-18

**Authors:** Daniel P. Judge, Julian D. Gillmore, Kevin M. Alexander, Amrut V. Ambardekar, Francesco Cappelli, Marianna Fontana, Pablo García-Pavía, Justin L. Grodin, Martha Grogan, Mazen Hanna, Ahmad Masri, Jose Nativi-Nicolau, Laura Obici, Steen Hvitfeldt Poulsen, Nitasha Sarswat, Keyur Shah, Prem Soman, Ted Lystig, Xiaofan Cao, Kevin Wang, Maria Lucia Pecoraro, Jean-François Tamby, Leonid Katz, Uma Sinha, Jonathan C. Fox, Mathew S. Maurer

**Affiliations:** Division of Cardiology, Medical University of South Carolina, Charleston (D.P.J.).; National Amyloidosis Centre, University College London, Royal Free Hospital, UK (J.D.G., M.F.).; Division of Cardiovascular Medicine, Department of Medicine, Stanford Center for Clinical Research, Stanford University School of Medicine, Palo Alto, CA (K.M.A.).; Department of Medicine, Division of Cardiology, Consortium for Fibrosis Research & Translation, University of Colorado Anschutz Medical Campus, Aurora (A.V.A.).; Tuscan Regional Amyloidosis Centre, Careggi University Hospital, Florence, IT (F.C.).; Department of Cardiology, Hospital Universitario Puerta de Hierro Majadahonda and Centro Nacional de Investigaciones Cardiovasculares, Madrid, ES (P.G.-P.).; Department of Internal Medicine, Division of Cardiology, University of Texas Southwestern Medical Center, Dallas (J.L.G.).; Department of Cardiovascular Medicine, Mayo Clinic, Rochester, MN (M.G.).; Department of Cardiovascular Medicine, Cleveland Clinic, OH (M.H.).; Division of Cardiology, Oregon Health & Science University, Portland (A.M.).; Department of Transplant, Mayo Clinic, Jacksonville, FL (J.N.-N.).; Amyloidosis Research and Treatment Centre, Fondazione IRCCS Policlinico San Matteo, Pavia, IT (L.O.).; Department of Cardiology, Aarhus University Hospital, DK (S.H.P.).; Division of Cardiovascular Medicine, University of Chicago Medicine, IL (N.S.).; Pauley Heart Center, Department of Cardiology, Virginia Commonwealth University, Richmond (K.S.).; Division of Cardiology, University of Pittsburgh Medical Center, PA (P.S.).; BridgeBio Pharma, Inc, San Francisco, CA (T.L., X.C., K.W., M.L.P., J.-F.T., L.K., U.S., J.C.F.).; Department of Medicine, Columbia University Irving Medical Center, New York, NY (M.S.M.).

**Keywords:** amyloidosis, familial, clinical trials as topic, exercise tolerance, hospitalization, mortality, quality of life, transthyretin-related heart failure

## Abstract

**BACKGROUND:**

In the phase 3 randomized controlled study ATTRibute-CM (Efficacy and Safety of AG10 in Subjects With Transthyretin Amyloid Cardiomyopathy), acoramidis, a transthyretin stabilizer, demonstrated significant efficacy on the primary end point. Participants with transthyretin amyloid cardiomyopathy who completed ATTRibute-CM were invited to enroll in an open-label extension study (OLE). We report the efficacy and safety data of acoramidis in participants who completed ATTRibute-CM and enrolled in the ongoing OLE.

**METHODS:**

Participants who previously received acoramidis through month 30 in ATTRibute-CM continued to receive it (continuous acoramidis), and those who received placebo through month 30 were switched to acoramidis (placebo to acoramidis). Participants who received concomitant tafamidis in ATTRibute-CM were required to discontinue it to be eligible to enroll in the OLE. Clinical efficacy outcomes analyzed through month 42 included time to event for all-cause mortality (ACM) or first cardiovascular-related hospitalization (CVH), ACM alone, first CVH alone, ACM or recurrent CVH, change from baseline in NT-proBNP (N-terminal pro-B-type natriuretic peptide), 6-minute walk distance, serum transthyretin, and Kansas City Cardiomyopathy Questionnaire Overall Summary score. Safety outcomes were analyzed through month 42.

**RESULTS:**

Overall, 438 of 632 participants in ATTRibute-CM completed treatment, and 389 enrolled in the ongoing OLE (263 continuous acoramidis and 126 placebo to acoramidis). The hazard ratio for ACM or first CVH was 0.57 (95% CI, 0.46–0.72) at month 42 based on a stratified Cox proportional hazards model (*P*<0.0001) favoring continuous acoramidis. Similar analyses were performed on ACM alone and first CVH alone, with hazard ratios of 0.64 (95% CI, 0.47–0.88) and 0.53 (95% CI, 0.41–0.69), respectively, at month 42. Treatment effects for NT-proBNP and 6-minute walk distance also favored continuous acoramidis. On initiation of open-label acoramidis in the placebo-to-acoramidis arm, there was a prompt increase in serum transthyretin. Quality of life assessed by Kansas City Cardiomyopathy Questionnaire Overall Summary score was well preserved in continuous-acoramidis participants compared with the placebo-to-acoramidis participants. No new clinically important safety issues were identified in this long-term evaluation.

**CONCLUSIONS:**

Early initiation and continuous use of acoramidis in the ATTRibute-CM study through month 42 of the ongoing OLE study were associated with sustained clinical benefits in a contemporary transthyretin amyloid cardiomyopathy cohort, with no clinically important safety issues newly identified.

**REGISTRATION:**

URL: https://www.clinicaltrials.gov; Unique identifier: NCT04988386.

**Clinical Perspective**

**What Is New?**

- This article extends the observations from the ATTRibute-CM trial (Efficacy and Safety of AG10 in Subjects With Transthyretin Amyloid Cardiomyopathy) of acoramidis in the treatment of transthyretin amyloid cardiomyopathy.
- ATTRibute-CM was designed with a fixed treatment duration of 30 months.
- The treatment effects on clinical outcomes (all-cause mortality and cardiovascular-related hospitalization) and other measures of morbidity, physical function, and quality of life are reported out to 42 months for participants continuously treated with acoramidis compared with those previously receiving placebo and initiating acoramidis in the open-label extension.

**What Are the Clinical Implications?**

- These long-term data support the positive efficacy and safety profile of acoramidis.
- Because participants who received concomitant tafamidis in ATTRibute-CM were required to discontinue tafamidis to be eligible for the open-label extension, these data from the open-label extension can be interpreted without the potential confounder of concomitant tafamidis use.
- Observations from the open-label extension suggest that reductions in functional capacity and quality of life observed in the placebo arm of ATTRibute-CM do not recover if disease-modifying therapy is delayed.
- Most important, the treatment effects of continuous acoramidis on clinical outcomes underscore the importance of early diagnosis and initiation of disease-modifying therapy.

Transthyretin amyloid cardiomyopathy (ATTR-CM) is a progressive cardiovascular disease characterized by destabilization and dissociation of transthyretin tetramers, misfolding and aggregation of unstable transthyretin monomers into toxic amyloid precursors, and their deposition into the myocardium, where they organize to form amyloid fibrils.^1,2^ Patients with ATTR-CM experience progressive heart failure symptoms of a restrictive cardiomyopathy, including worsening functional capacity, lower quality of life, and an increased risk of premature death.^1–3^ Despite recent improvements in disease awareness, earlier diagnosis, and better management of background therapy,^4–6^ delays in diagnosis and resulting impairments in quality of life are common.^3,7^

Acoramidis is an investigational, near-complete (≥90%) transthyretin stabilizer when administered at the regimen used in both ATTRibute-CM (Efficacy and Safety of AG10 in Subjects With Transthyretin Amyloid Cardiomyopathy) and the ongoing open-label extension (OLE).^8–10^ The efficacy and safety of acoramidis were evaluated for 30 months in the randomized, double-blind, placebo-controlled phase 3 ATTRibute-CM trial.^8^ In ATTRibute-CM, acoramidis demonstrated significant efficacy on the hierarchical, sequentially analyzed, combined end point of all-cause mortality (ACM), frequency of cardiovascular-related hospitalization (CVH), change from baseline in NT-proBNP (N-terminal brain-type natriuretic peptide), and change from baseline in 6-minute walk distance (6MWD).^8^ At month 30, compared with placebo, acoramidis also demonstrated benefits in components of the primary end point and other end points, including a 36% risk reduction for the time to ACM or first CVH with effects observed after 3 months of treatment,^11^ a 50% risk reduction in the annualized frequency of CVH,^8^ and a 42% risk reduction in ACM or recurrent CVH as analyzed with a negative binomial regression method.^12^ In patients with ATTR-CM, 30 months of treatment with acoramidis reduced the rate of disease progression as assessed by NT-proBNP and better preserved the overall quality of life as assessed by the Kansas City Cardiomyopathy Questionnaire Overall Summary (KCCQ-OS) compared with placebo.^8^

The primary objective of the OLE is to collect and analyze long-term safety data in this study population. Secondary objectives include continuing to monitor and chart clinical outcomes (ACM, CVH) and associated measures of morbidity, physical function, and quality of life.

## METHODS

### Data Availability Statement

Qualified academic investigators may submit requests for access to data at MedInfo@BridgeBio.com. Requests for access to study data will be evaluated by BridgeBio Pharma, Inc, and access will be provided contingent on the approval of a research or study proposal and the execution of a data sharing agreement. BridgeBio Pharma will consider requests for access to participant-level data if participant privacy is ensured through methods such as data deidentification, pseudonymization, or anonymization (as required by applicable law) and if such disclosures were included in the informed consent form or study protocol of the relevant study.

### Study Design and Participants

This is an open-label extension (URL: www.clinicaltrials.gov; Unique identifier: NCT04988386) of a phase 3, multicenter, double-blind, randomized, placebo-controlled trial, ATTRibute-CM (URL: www.clinicaltrials.gov; Unique identifier: NCT03860935). The study protocol was approved at participating sites by their respective research ethics committees. The trial continues in accordance with the International Council for Harmonisation, Good Clinical Practice, and the Declaration of Helsinki. All participants provided written informed consent.

Participants who completed treatment in the 30-month ATTRibute-CM study of acoramidis versus placebo and met OLE eligibility criteria (Figure S1) were invited to participate in the OLE study. Although ATTRibute-CM allowed initiation of tafamidis at the discretion of the investigator after 12 months, the use of tafamidis is not allowed in the OLE. Therefore, participants on tafamidis at the end of the ATTRibute-CM study who elected to enroll in OLE were required to discontinue it before starting the OLE. For the results in this article, we continue to analyze participants according to the 2 original randomization groups (acoramidis and placebo) from ATTRibute-CM. Of the 438 participants who completed treatment in ATTRibute-CM, 49 elected not to enroll in the OLE (34 from the acoramidis group and 15 from the placebo group). The most common reason was related to tafamidis treatment (ie, choosing to continue tafamidis if they received it as a concomitant medication during ATTRibute-CM or choosing to initiate tafamidis treatment on completing ATTRibute-CM). All participants in the OLE received acoramidis HCl 800 mg twice daily. Participants who had previously received acoramidis for 30 months in ATTRibute-CM continued to receive it in the OLE (continuous acoramidis), and participants who had received placebo for 30 months in ATTRibute-CM were switched from placebo to open-label acoramidis treatment (placebo to acoramidis). Participants in the OLE study returned for planned study visits at 1 month, at 6 months, and every 6 months thereafter. This report contains data accumulated through month 12 of the OLE (month 42 since randomization in ATTRibute-CM) for all OLE participants.

### Outcomes, End Points, and Statistical Analyses

Observations were analyzed comparing the continuous-acoramidis and placebo-to-acoramidis cohorts. Clinical end points (ACM and CVH) were defined as for ATTRibute-CM.^8^ CVH events included all CVHs observed through month 42 for participants continuing treatment and up to 30 days after early treatment discontinuation. All ACM and investigator-identified CVH events in the OLE study were adjudicated by a Clinical Event Committee (as in ATTRibute-CM) without knowledge of prior treatment assignment in ATTRibute-CM. Adjudicated events were the basis for the efficacy analyses of ACM and CVH.

Time-to-event analyses were performed with a stratified Cox proportional hazards model that included treatment group as an explanatory factor and baseline 6MWD as a covariate and stratified by the ATTRibute-CM randomization stratification factors of genotype, NT-proBNP level, and estimated glomerular filtration rate (eGFR). The proportional hazards assumption was checked through examination of both Schoenfeld and martingale residuals; no major violations of the proportional hazards assumption were observed for either the time-to-first-CVH model or the time-to-first-CVH or -ACM model. The deviations in assumptions for the ACM alone model reported previously^8^ appear to continue, but we report the results to provide relevant context for all of the findings. Analyses are reported for data through month 42. Analyses included time to event for ACM or first CVH, ACM alone, and first CVH alone. Kaplan-Meier curves by treatment group were plotted for these analyses. Comparable analyses were examined with data through month 36 to assess the constancy of treatment effect over varying follow-up time.

The annualized frequency of cumulative ACM or recurrent CVH events was analyzed using a negative binomial regression model with treatment group, the randomization stratification factors applied in ATTRibute-CM (genotype, NT-proBNP level, and eGFR level), and an offset term of the logarithm of the follow-up duration. Changes from baseline in NT-proBNP and 6MWD are summarized descriptively, and the mean (geometric mean fold change for NT-proBNP) with error bars for the change from baseline values over time are presented. Serum transthyretin, an in vivo surrogate of transthyretin stabilization, was also analyzed as change from baseline (at entry to the OLE) in the 2 cohorts. Quality of life was assessed serially by the KCCQ-OS score. Safety was assessed by tabulating treatment-emergent adverse events according to frequency, seriousness, severity, relatedness to study treatment, and discontinuation of study drug.

### Trial Oversight, Independent Data Access, and Analysis

An independent data monitoring committee that monitored unblinded data throughout the ATTRibute-CM study continues to monitor the long-term safety of acoramidis in the OLE. The sponsor designed and conducted the statistical analyses for the OLE according to a prespecified statistical analysis plan. Data continue to be entered electronically, and all authors have full access to the study data at this initial data freeze, yielding the data for this report.

## RESULTS

### Baseline Characteristics and Demographics

The baseline characteristics were well balanced among the 632 participants who were randomized 2:1 (acoramidis:placebo) in the ATTRibute-CM trial.^8^ For 30 months in ATTRibute-CM, participants in the acoramidis group experienced treatment-related benefits, whereas participants in the placebo group experienced a greater degree of disease progression. A total of 438 participants completed treatment in ATTRibute-CM. Of these participants, a total of 389 (89%) met eligibility criteria, including a willingness to discontinue concomitant open-label tafamidis if taken during ATTRibute-CM, and were enrolled in the OLE (Figure S2). Of those, 263 participants who received acoramidis in the ATTRibute-CM trial continued to receive acoramidis in the OLE (continuous acoramidis), and 126 participants who received placebo in ATTRibute-CM switched to open-label acoramidis (placebo to acoramidis). Their baseline characteristics and demographics at entry to the OLE are summarized in Table 1. Of particular importance are parameters associated with progression of disease or predictive of mortality (New York Heart Association class, NT-proBNP levels, National Amyloidosis Centre stage, and serum transthyretin levels).

**Table 1.**
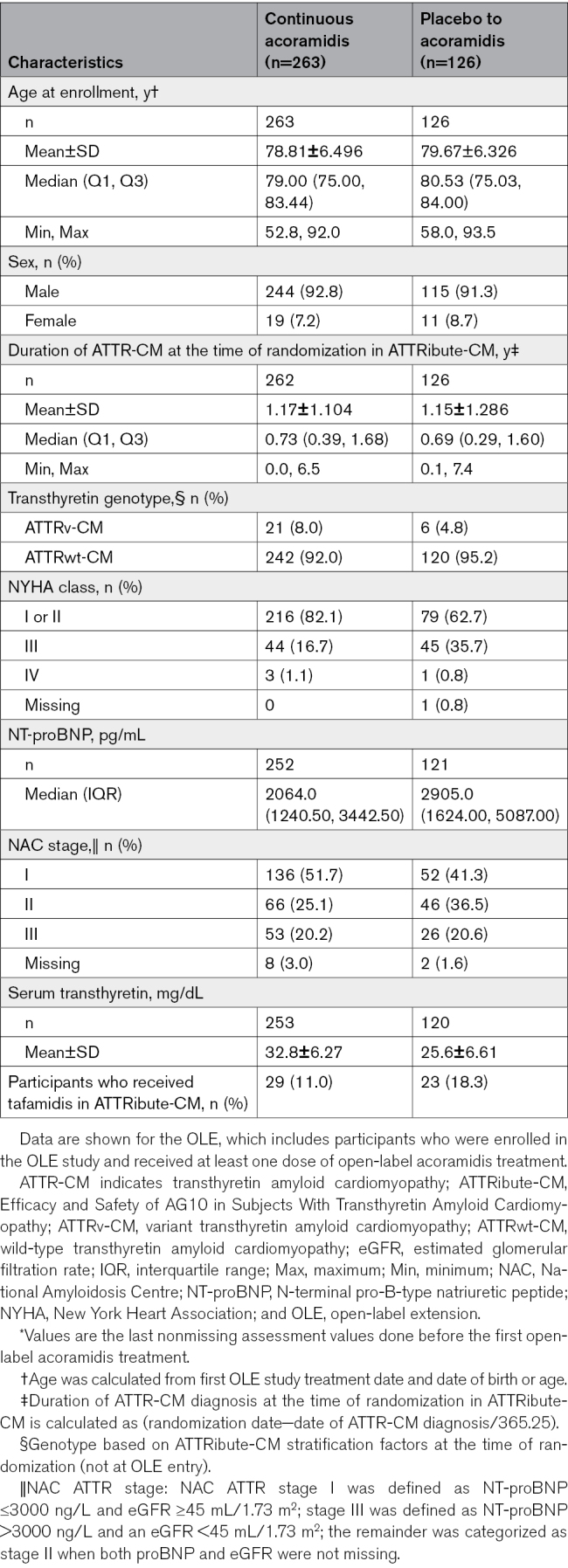
Demographics and Characteristics of Participants at Entry^*^ to the OLE

### Clinical Outcomes of ACM and CVH

**Figure 1.**
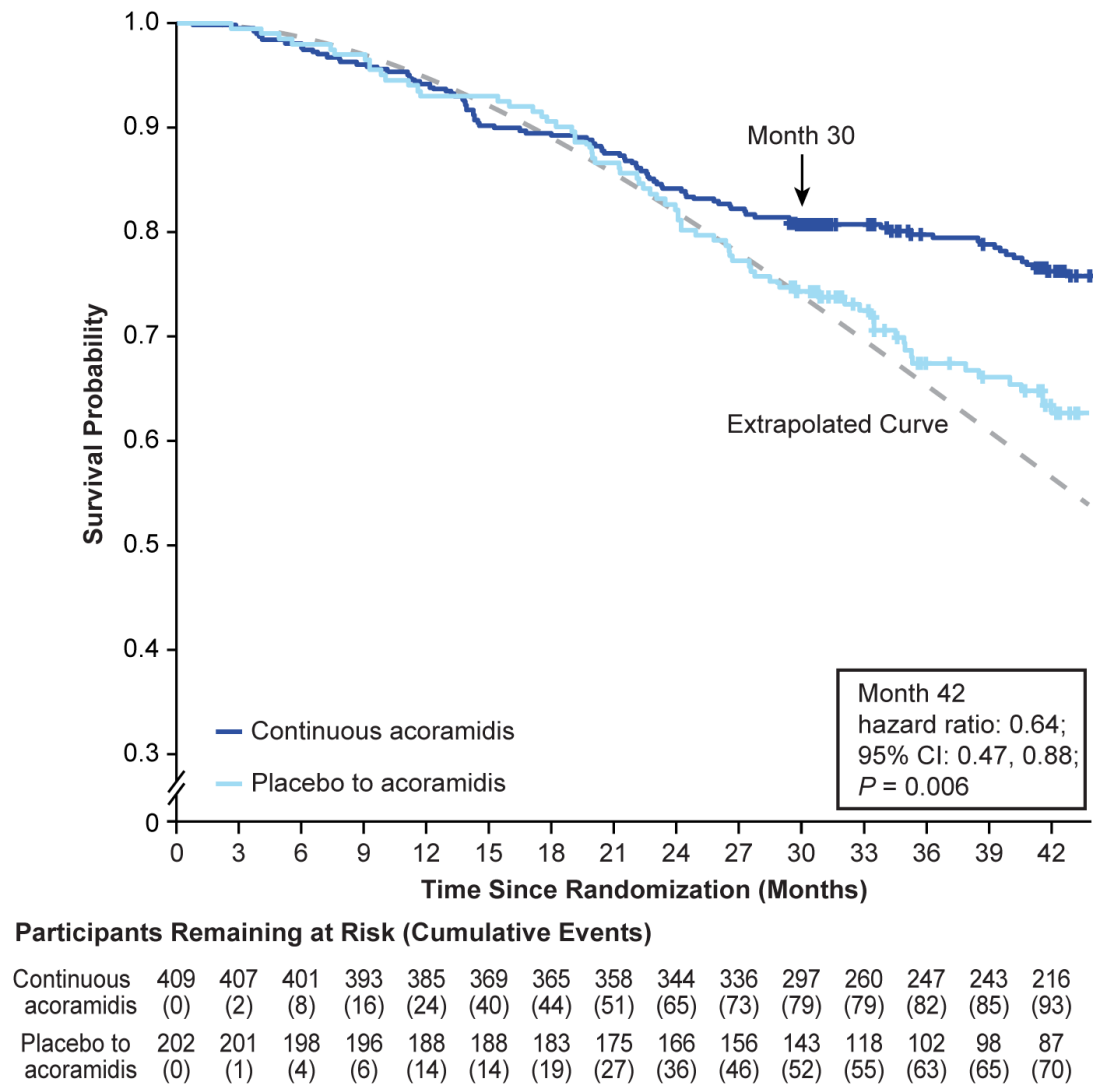
**Kaplan-Meier curve for time to all-cause mortality from baseline in ATTRibute-CM study through month 42 in the OLE study.** Data are for the full analysis set. The full analysis set included the modified intention-to-treat population in ATTRibute-CM (Efficacy and Safety of AG10 in Subjects With Transthyretin Amyloid Cardiomyopathy), which was defined as all participants who were randomized to acoramidis or placebo, received ≥1 dose of acoramidis or placebo, had ≥1 efficacy evaluation after baseline, and had a baseline estimated glomerular filtration rate (eGFR) of ≥30 mL/1.73 m^2^. The arrow at month 30 indicates the final follow-up time point in ATTRibute-CM and the beginning of the open-label extension (OLE) study. Data were analyzed with a stratified Cox proportional hazards model that included treatment group as an explanatory factor and baseline 6-minute walk distance as a covariate and stratified by the ATTRibute-CM randomization stratification factors of genotype, NT-proBNP (N-terminal pro-B-type natriuretic peptide), and eGFR. The extrapolated curve shows expected results if participants had continued receiving placebo in the OLE study. Survival probabilities for placebo to acoramidis treatment group beyond month 30, assuming that no open-label acoramidis had been taken, were extrapolated according to a Weibull probability model for the time to the all-cause mortality event estimated from the data observed in the ATTRibute-CM study and represented by the dotted line.

The percentage of participants with ACM was reduced with continuous acoramidis compared with placebo to acoramidis at month 42 (23.0% [94/409] versus 34.7% [70/202]), corresponding to a relative risk reduction of 33.7%. With the use of a stratified Cox proportional hazards model, the hazard ratio for ACM through month 42 from initiation of therapy in ATTRibute-CM was 0.64 (95% CI, 0.47–0.88; *P*=0.006; Figure 1). Results through months 36 and 42 are shown in Table S1.

**Figure 2.**
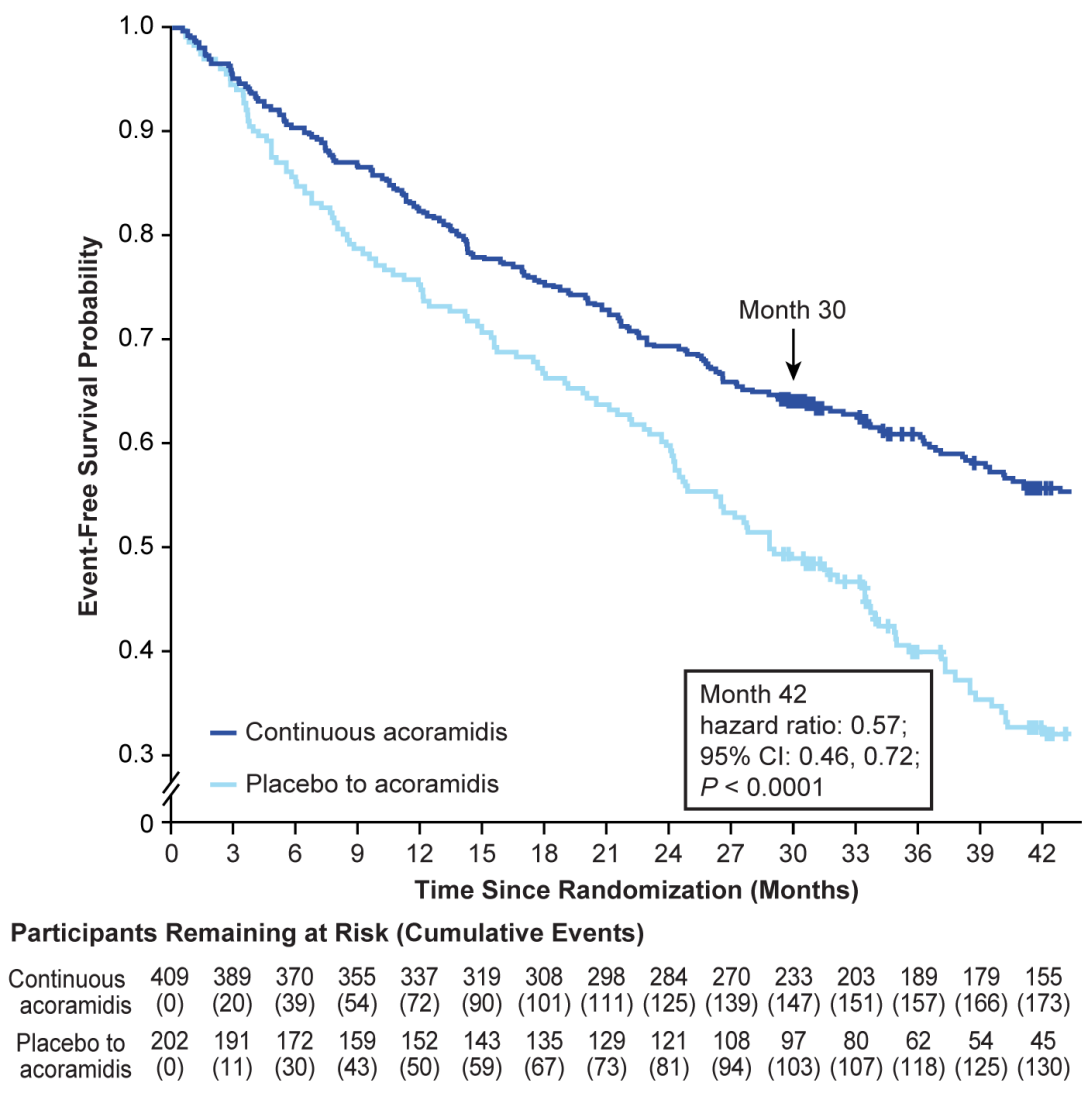
**Kaplan-Meier curve for time to all-cause mortality or first cardiovascular-related hospitalization from baseline in ATTRibute-CM through month 42 in the OLE study.** Data are for the full analysis set. The full analysis set included the modified intention-to-treat population in ATTRibute-CM (Efficacy and Safety of AG10 in Subjects With Transthyretin Amyloid Cardiomyopathy), which was defined as all participants who were randomized to acoramidis or placebo, received ≥1 dose of acoramidis or placebo, had ≥1 efficacy evaluation after baseline, and had a baseline estimated glomerular filtration rate (eGFR) of ≥30 mL/1.73 m^2^. The arrow at month 30 indicates the final follow-up time point in ATTRibute-CM and the beginning of the open-label extension (OLE) study. Data were analyzed with a stratified Cox proportional hazards model that included treatment group as an explanatory factor and baseline 6-minute walk distance as a covariate and stratified by the ATTRibute-CM randomization stratification factors of genotype, NT-proBNP (N-terminal pro-B-type natriuretic peptide), and eGFR.

ACM or first CVH was reported in 174 of 409 participants (42.5%) in the continuous-acoramidis group and 130 of 202 participants (64.4%) in the placebo-to-acoramidis group at month 42, corresponding to a 33.9% relative risk reduction. The hazard ratio for time to event for ACM or first CVH was 0.57 (95% CI, 0.46–0.72; *P*<0.0001) at month 42, based on a stratified Cox proportional hazards model favoring continuous acoramidis (Figure 2).

**Figure 3.**
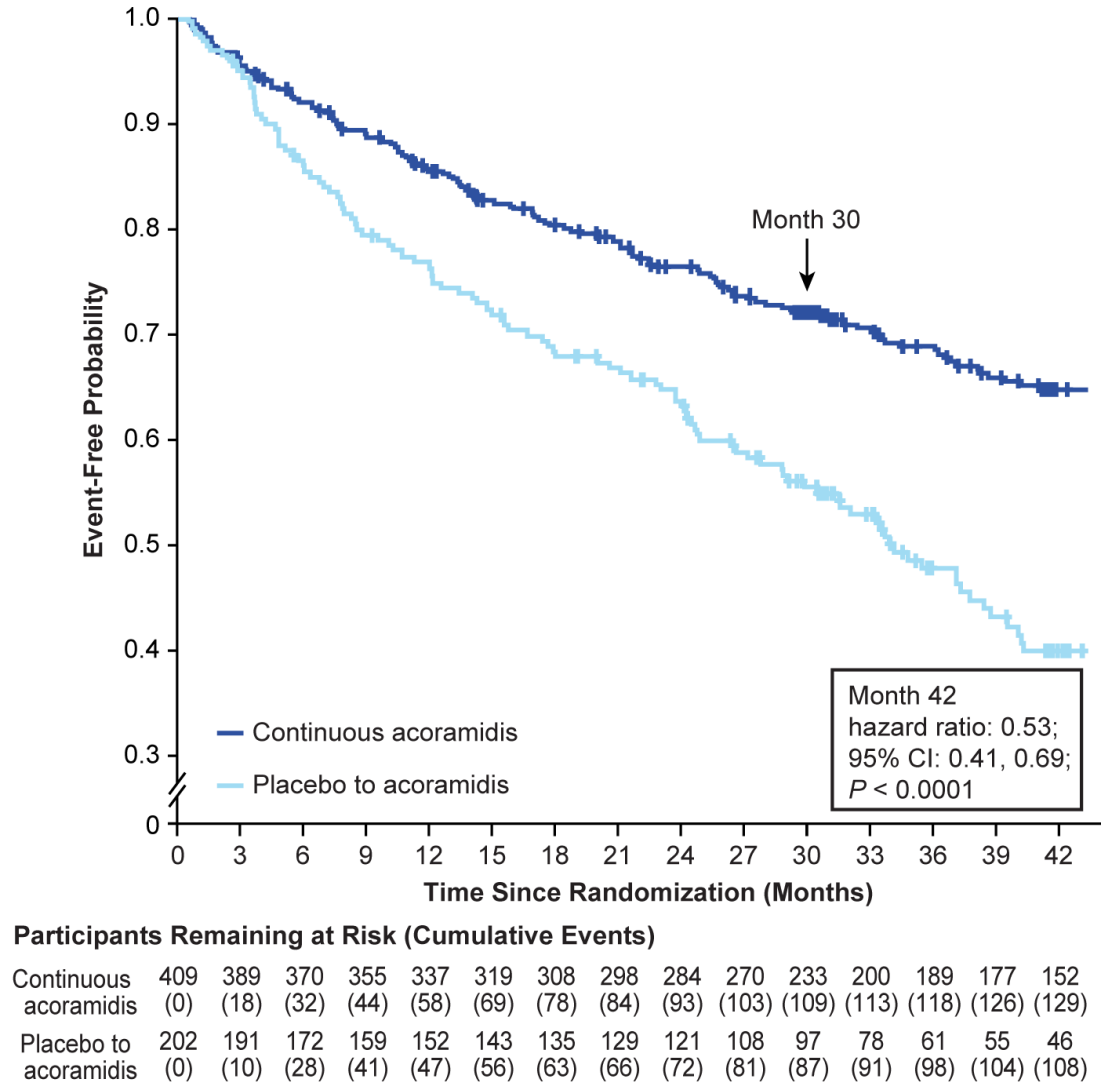
**Kaplan-Meier curve for time to first cardiovascular-related hospitalization from baseline in ATTRibute-CM through month 42 in the OLE study.** Data are for the full analysis set. The full analysis set included the modified intention-to-treat population in ATTRibute-CM (Efficacy and Safety of AG10 in Subjects With Transthyretin Amyloid Cardiomyopathy), which was defined as all participants who were randomized to acoramidis or placebo, received ≥1 dose of acoramidis or placebo, had ≥1 efficacy evaluation after baseline, and had a baseline estimated glomerular filtration rate (eGFR) rate of ≥30 mL/1.73 m^2^. The arrow at month 30 indicates the final follow-up time point in ATTRibute-CM and the beginning of the open-label extension (OLE) study. Data were analyzed with a stratified Cox proportional hazards model that included treatment group as an explanatory factor and baseline 6-minute walk distance as a covariate and stratified by the ATTRibute-CM randomization stratification factors of genotype, NT-proBNP (N-terminal pro-B-type natriuretic peptide), and eGFR.

A total of 129 of 409 participants (31.5%) in the continuous-acoramidis group and 108 of 202 participants (53.5%) in the placebo-to-acoramidis group reported CVH events through month 42, corresponding to a 41.0% relative risk reduction (Table S1). The hazard ratio for time to first CVH was 0.53 (95% CI, 0.41–0.69; *P*<0.0001), based on a stratified Cox proportional hazards model favoring continuous acoramidis (Figure 3).

The negative binomial regression analysis of the annualized frequency of cumulative ACM or recurrent CVH events showed that continuous acoramidis treatment led to a reduction in the relative risk of ACM or recurrent CVH by 48.2% through month 42 (relative risk ratio, 0.52 [95% CI, 0.39–0.68]; *P*<0.0001) compared with the placebo-to-acoramidis arm (Table 2).

**Table 2.**
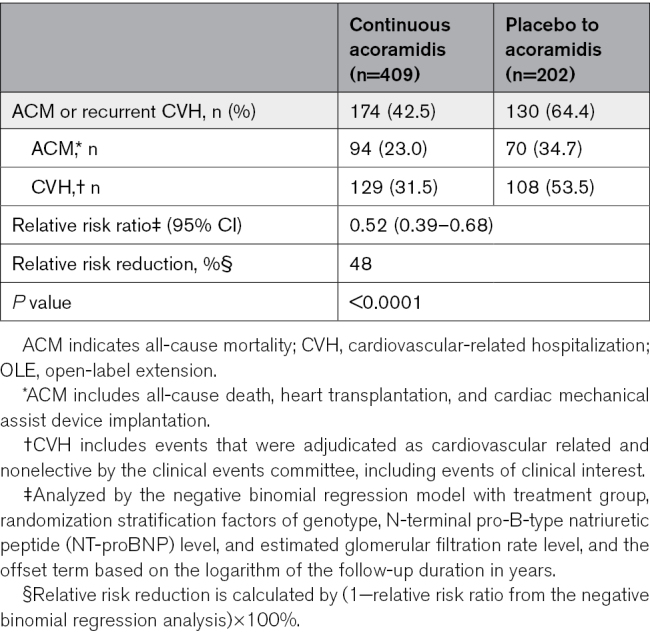
Summary of All-Cause Mortality or Recurrent Cardiovascular-Related Hospitalization Through Month 42 in the OLE Study

### Biomarkers, Functional Assessments, and Quality of Life

**Figure 4.**
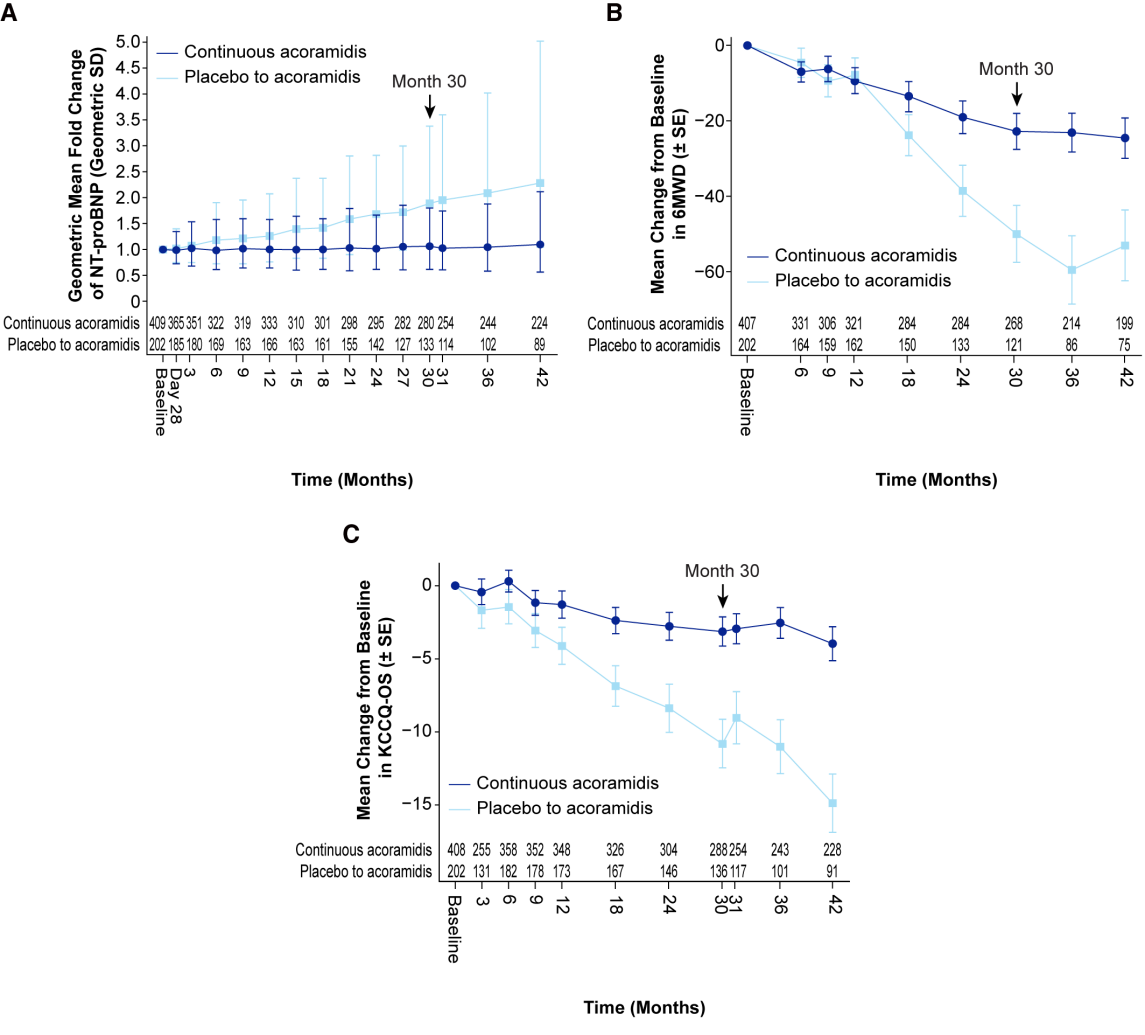
**Change from baseline in (A) geometric mean of fold change in NT-proBNP, (B) 6MWD, and (C) KCCQ-OS (observed values).** Data are for the full analysis set. The full analysis set included the modified intention-to-treat population in ATTRibute-CM (Efficacy and Safety of AG10 in Subjects With Transthyretin Amyloid Cardiomyopathy), which was defined as all participants who were randomized to acoramidis or placebo, received ≥1 dose of acoramidis or placebo, had ≥1 efficacy evaluation after baseline, and had a baseline estimated glomerular filtration rate (eGFR) of ≥30 mL/1.73 m^2^. The arrow at month 30 indicates the final follow-up time point in ATTRibute-CM and the beginning of the open-label extension study. 6MWD indicates 6-minute walk distance; KCCQ-OS, Kansas City Cardiomyopathy Questionnaire Overall Score; and NT-proBNP, N-terminal pro-B-type natriuretic peptide.

Key biomarker assessments included the change in NT-proBNP and serum transthyretin levels. An early separation in change from baseline in NT-proBNP that was observed in ATTRibute-CM continued into the OLE (Figure 4A). At month 42, the geometric mean (geometric SD) for fold change from baseline in NT-proBNP was 1.10 (1.93) in the continuous-acoramidis group and 2.29 (2.19) in the placebo-to-acoramidis group. In the placebo-to-acoramidis arm, there may be a flattening of the slope in the NT-proBNP trajectory, but the number of participants with available assessments was small, and additional follow-up is required to clarify any incipient treatment effect of acoramidis in the placebo-to-acoramidis group.

An early increase (by day 28) in serum transthyretin (an in vivo reflection of transthyretin stabilization) observed in ATTRibute-CM was sustained in the continuous-acoramidis arm through month 30 and did not change in the first month of the OLE (month 31 since randomization in ATTRibute-CM).^13^ In the continuous-acoramidis arm, mean±SE change from baseline in serum transthyretin was 9.1±0.34 mg/dL at month 30 and 8.9±0.38 mg/dL at month 31. In the placebo-to-acoramidis arm, mean±SE change from baseline in serum transthyretin was 1.3±0.55 mg/dL at month 30 and 7.4±0.55 mg/dL at month 31.^13^

Functional capacity was assessed by the standardized 6-minute walk test and analyzed by change from baseline in distance achieved (6MWD). In ATTRibute-CM, a treatment effect on relative preservation of 6MWD was observed starting after month 12 with a statistically significant difference assessed at month 30. This trend continued into the OLE with mean±SE change from baseline in distance walked in the continuous acoramidis arm of –24.5±5.33 m at month 42 (Figure 4B).

An early separation in change from baseline in KCCQ-OS score observed in ATTRibute-CM continued into the OLE (Figure 4C). At 42 months in the continuous-acoramidis arm, mean±SE change from baseline in KCCQ-OS score was –4.0±1.15.

### Safety Summary

In participants continuing acoramidis in the OLE, the overall safety assessment reflects the progressive nature of the disease under study, comorbidities expected for this population, demographic profile of the participants, and duration of investigation. No new clinically important safety issues were identified in this long-term evaluation of acoramidis up to 42 months (Table 3).

**Table 3.**
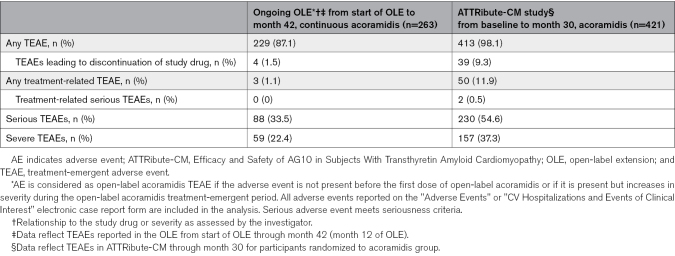
Treatment-Emergent Adverse Events in the Continuous Acoramidis Group to Month 42 in the OLE^*†^ and Acoramidis Group to Month 30 in ATTRibute-CM^†^

## DISCUSSION

The extended observation period of the OLE in this initial report covers 42 months of continuous treatment from the initiation of acoramidis treatment in ATTRibute-CM. The previously observed positive trend in the treatment effect of acoramidis on ACM continued and was statistically significant at 42 months of treatment compared with the cohort of participants who switched to acoramidis from placebo on entry to the OLE. We note that the clinical characteristics of the participants at entry to the OLE reflect both the treatment effect of early and continuous acoramidis treatment in the active treatment arm of ATTRibute-CM and the continued disease progression in those participants in the placebo arm of ATTRibute-CM. Although the characteristics of the 2 arms at the initiation of ATTRibute-CM were well balanced and reflective of a contemporary ATTR-CM population, the imbalance observed in some clinical parameters at the entry to the OLE (eg, New York Heart Association class distribution, National Amyloidosis Centre stage distribution, and NT-proBNP levels) highlights the progression of disease in the ATTRibute-CM placebo arm despite improvements in background clinical management of heart failure in ATTR-CM. This almost certainly influenced the subsequent behavior with respect to the response to acoramidis in the 2 arms of the OLE. In addition, the fact that the placebo-to-acoramidis participants had only been treated with acoramidis for 12 months at the time of this analysis could contribute to continued progression in that group compared with the continuous-acoramidis group. When we extrapolated the Kaplan-Meier curve observed in the placebo-to-acoramidis arm up to month 30 in ATTRibute-CM (Figure 1), we observed that there may be a trend of reduction in the risk of ACM in this arm after initiation of acoramidis in the OLE as observed out to month 42. Although the trajectory of events accruing over time in the placebo-to-acoramidis group appears to persist from month 30, additional follow-up in this ongoing study may clarify the currently observed favorable impact of acoramidis treatment in this group.

Clinical outcomes analyzed with the more commonly used time-to-event approach of the clinical outcome of ACM or first CVH when extended to month 42 confirm the robust and persistent treatment effect that was observed as early as month 3 in ATTRibute-CM (Figure 2; Table S1). We evaluated this in a similar analysis of first CVH alone that also confirms the robustness and persistence of the treatment effect on CVH observed in ATTRibute-CM through 42 months of continuous acoramidis treatment in the OLE.

The time-to-event analyses, unlike the win ratio and Finkelstein-Schoenfeld test used for the primary end point in ATTRibute-CM, do not account for recurrent CVH events. We include in this report results from a negative binomial regression analysis to document the robust and sustained treatment effect of acoramidis not only on ACM and first CVH but also on recurrent CVH events. Taken together, these analyses of clinical outcomes demonstrate consistency across several different analytical approaches to the ATTRibute-CM trial and its OLE. The exclusion from the OLE of participants who received open-label tafamidis in ATTRibute-CM was nonrandomized and reduced the potential participation of patients who otherwise completed treatment in ATTRibute-CM and would have been eligible to enter the OLE except for their decision to not participate (mainly to continue open-label treatment with tafamidis). Although this is a potential limitation of the OLE (see below), it removes a potential confounder from the long-term data on the efficacy and safety of acoramidis.

We included analyses of the prognostic biomarker NT-proBNP and an assessment of functional capacity (6MWD) in the OLE to extend the observations from ATTRibute-CM. Findings of continued suppression of a progressive increase in NT-proBNP and relative preservation of functional capacity by 6MWD extend the robust treatment effect of acoramidis to 42 months of treatment.

Consistent with the early separation in the trajectory of the treatment effect of acoramidis on CVH at month 3, early separation was also observed in NT-proBNP between months 3 and 6 and in quality of life as assessed by KCCQ-OS score by month 3. Given the limited evidence from a single study, any consideration of a potential mechanism of how these early treatment effects might be interrelated would be speculative, but the observation identifies an area for additional investigation.

Acoramidis has been established through multiple lines of evidence to be a near-complete (≥90%) transthyretin stabilizer when administered at the regimen used in ATTRibute-CM and its OLE,^8–10,14^ so the observed increase in serum transthyretin (an in vivo reflection of transthyretin stabilization) in the participants initiating acoramidis in the OLE is not surprising. The magnitude of the increase observed in the placebo-to-acoramidis arm at month 1 of the OLE is consistent with the initial observation at day 28 of ATTRibute-CM in participants randomized to acoramidis.^8^ In separate analyses not reported here, we have demonstrated that the increase in serum transthyretin after initiation of acoramidis treatment is predictive of survival in ATTR-CM (Mathew S. Maurer, MD, et al, unpublished data, 2024).

The primary objective of the OLE study is to continue collecting long-term safety information beyond the 30-month fixed duration of treatment in the ATTRibute-CM study.^8^ No new clinically important safety issues were identified in this long-term evaluation of acoramidis up to 42 months. Although the relative frequency of adverse events cannot be ascertained in the absence of a control group, in participants continuing acoramidis in the OLE, the overall safety assessment reflects the progressive nature of the disease under study, comorbidities expected for this population, demographic profile of the participants, and duration of investigation.

This report demonstrates consistency across efficacy and safety observations and analyses that support the potential for acoramidis to make an important contribution to the expanding pharmacopoeia available to physicians for the treatment of their patients with ATTR-CM.

### Limitations of the Study

Although the OLE is by definition unblinded and lacks a true control arm, the trajectories of the effects of continuous acoramidis treatment observed out to 42 months from initiation of therapy in ATTRibute-CM underscore the importance of early and continuous administration of disease-modifying treatment. Participants in ATTRibute-CM who completed treatment to 30 months but did not elect to participate in the OLE reduce the power of the estimates of the treatment effect, which may have been influenced by the nonrandom nature of who chose to continue in the OLE. Although a potential limitation, the exclusion of participants who received open-label tafamidis in ATTRibute-CM from the OLE removes a potential confounder from the interpretation of the analyses presented in this report. Because participants who received acoramidis for 30 months in ATTRibute-CM derived a treatment benefit while those who received placebo experienced a greater degree of disease progression, the baseline characteristics of participants in the 2 arms of the OLE were not balanced, especially for parameters associated with disease progression, which may have influenced the estimated benefits of acoramidis treatment in the OLE. Last, the open-label design also conveys unavoidable uncertainty about the interpretation of long-term safety data without a true control group for comparison.

### Conclusions

This initial report from an ongoing OLE of the ATTRibute-CM study^8^ of acoramidis for the treatment of ATTR-CM adds clinically important observations of longer-term safety and efficacy than was available at the conclusion of the ATTRibute-CM study. In particular, the extended follow-up demonstrates a reduced risk of ACM that is highly statistically significant, with consistently significant beneficial treatment effects on the entire panel of end points analyzed alone or together and with different analytical methods. Together, these data continue to support the potential for acoramidis to make an important contribution to the care of patients with ATTR-CM.

## ARTICLE INFORMATION

### Acknowledgments

The authors thank the participating patients, their families, and the caregivers, investigators, and study center staff for their participation. All authors were involved in writing the manuscript and critically reviewed and revised the manuscript for submission. The authors vouch for the data and their analyses, and the sponsor of the study (BridgeBio Pharma, Inc) backs the fidelity of this report to the study protocol. The authors thank Jing Du, MD, MS, from BridgeBio Pharma, Inc for helping with additional statistical analyses. The authors thank Julie Hoegi, MD, from BridgeBio Pharma, Inc for helping with safety analyses. Under guidance from the authors, Michael Dyle, PhD, of Oxford PharmaGenesis provided additional medical writing assistance, which was funded by BridgeBio Pharma, Inc. Editorial support and critical review were provided by Shweta Rane, PhD, CMPP, and Souhiela Fawaz, PhD, of BridgeBio Pharma, Inc. The interpretation of the data and the decision to submit the manuscript to the journal were done independently by the authors without any influence from the sponsor of the study.

### Sources of Funding

The ATTRibute-CM and OLE studies were funded by BridgeBio Pharma, Inc, San Francisco, CA.

### Disclosures

Dr Judge has received consultancy fees from Alexion Pharmaceuticals, Alleviant Medical, Alnylam Pharmaceuticals, Attralus, BridgeBio Pharma, Cytokinetics, Lexeo Therapeutics, Novo Nordisk, Pfizer, Rocket Pharmaceuticals, Renovacor, and Tenaya Therapeutics; his institution received clinical trial funding from Array Biopharma, BridgeBio Pharma (formerly Eidos Therapeutics), MyoKardia, and Pfizer. Dr Gillmore has acted as a consultant, advisor, or speaker for Alnylam Pharmaceuticals, AstraZeneca, Attralus, BridgeBio Pharma, Intellia Therapeutics, Ionis Pharmaceuticals, Lycia Therapeutics, and Pfizer. Dr Alexander has acted as a consultant, advisor, or speaker for Arbor Biotechnologies, Attralus, Intellia Therapeutics, and Prothena Biosciences. Dr Ambardekar reports no conflicts. Dr Cappelli has acted as a consultant, advisor, or speaker for Alnylam Pharmaceuticals, Amicus Therapeutics, AstraZeneca, BridgeBio Pharma (formerly Eidos Therapeutics), Novo Nordisk, Bayer, and Pfizer. Dr Fontana has acted as a consultant, advisor, or speaker for Alnylam, Alexion, AstraZeneca, Attralus, Bayer, BridgeBio Pharma (formerly Eidos Therapeutics), Caelum Biosciences, Cardior, Intellia Therapeutics, Ionis Pharmaceuticals, Janssen Pharmaceuticals, Lexeo Therapeutics, Novo Nordisk, Pfizer, and Prothena Biosciences; owns stock or stock options of Lexeo Therapeutics and Myocardium; has received research grants from Alnylam, AstraZeneca, BridgeBio Pharma, and Pfizer; and has received a salary from British Heart Foundation intermediate fellowship. Dr García-Pavía has acted as a consultant, advisor, or speaker for Alexion Pharmaceuticals, Alnylam Pharmaceuticals, AstraZeneca, Attralus, Bayer, BridgeBio Pharma (formerly Eidos Therapeutics), Intellia Therapeutics, Ionis Pharmaceuticals, Neuroimmune, Novo Nordisk, and Pfizer; his institution received research or educational funding from Alnylam Pharmaceuticals, AstraZeneca, BridgeBio Pharma (formerly Eidos Therapeutics), Intellia Therapeutics, Novo Nordisk, and Pfizer. Dr Grodin has acted as a researcher for the Texas Health Resources clinical scholarship, BridgeBio Pharma (formerly Eidos Therapeutics), Pfizer, and National Heart, Lung, and Blood Institute (R01HL160892) and as a consultant, advisor, or speaker for Alexion, Alnylam, AstraZeneca, BridgeBio Pharma, Intellia, Lumanity, Novo Nordisk, Pfizer, Tenax Therapeutics, and Ultromics. Dr Grogan has acted as a researcher for Alnylam Pharmaceuticals, BridgeBio Pharma (formerly Eidos Therapeutics), Intellia Therapeutics, Janssen Pharmaceuticals, Novo Nordisk, and Pfizer and as a consultant, advisor, or speaker for Alnylam, BridgeBio Pharma, Janssen Pharmaceuticals, Novo Nordisk, and Pfizer. Dr Hanna has acted as a consultant, advisor, or speaker for Alexion Pharmaceuticals, Alnylam Pharmaceuticals, Attralus, BridgeBio Pharma (formerly Eidos Therapeutics), Ionis Pharmaceuticals, and Pfizer. Dr Masri has acted as a researcher for Attralus, Cytokinetics, Ionis Pharmaceuticals, and Pfizer and as a consultant, advisor, or speaker for Akros Pharma, Alexion Pharmaceuticals, Alnylam Pharmaceuticals, AstraZeneca, Attralus, BioMarin Pharmaceutical, BridgeBio Pharma (formerly Eidos Therapeutics), Bristol Myers Squibb, Cytokinetics, Haya Therapeutics, Ionis Pharmaceuticals, Lexicon Pharmaceuticals, Pfizer, Prothena Biosciences, and Tenaya Therapeutics. Dr Nativi-Nicolau has acted as a researcher for Alnylam Pharmaceuticals, AstraZeneca, BridgeBio Pharma (formerly Eidos Therapeutics), and Pfizer. Dr Obici has acted as a consultant, advisor, or speaker for Alnylam Pharmaceuticals, AstraZeneca, BridgeBio Pharma (formerly Eidos Therapeutics), Ionis Pharmaceuticals, Novo Nordisk, Pfizer, and Sobi–Swedish Orphan Biovitrum. Dr Hvitfeldt Poulsen has received consulting fees from Bayer A/S, BridgeBio Pharma (formerly Eidos Therapeutics), and Pfizer A/S and research support from Novo Nordisk A/S. Dr Sarswat has acted as a researcher for Pfizer and as a consultant, advisor, or speaker for Alnylam, AstraZeneca, BridgeBio Pharma (formerly Eidos Therapeutics), Novo Nordisk, and Pfizer. Dr Shah has acted as a consultant, advisor, or speaker for Pfizer. Dr Soman has acted as a researcher for Pfizer and as a consultant, advisor, or speaker for Alnylam Pharmaceuticals, BridgeBio Pharma (formerly Eidos Therapeutics), and Pfizer. Drs Lystig, Cao, Wang, Pecoraro, Tamby, Katz, Sinha, and Fox are employees and stockholders of BridgeBio Pharma.

Dr Maurer has acted as a researcher for the National Institutes of Health (R01HL139671 and R01AG081582-01), Alnylam Pharmaceuticals, Attralus, BridgeBio Pharma (formerly Eidos Therapeutics), Intellia Therapeutics, Ionis Pharmaceuticals, and Pfizer and as a consultant or advisor for Akcea Therapeutics, Alnylam Pharmaceuticals, AstraZeneca, Attralus, BridgeBio Pharma (formerly Eidos Therapeutics), Intellia Therapeutics, Ionis Pharmaceuticals, Novo Nordisk, and Pfizer.

### Supplemental Material

Figures S1 and S2

Table S1

## Supplementary Material


