# Supplementary material for: Long-Term Efficacy and Safety of Acoramidis in ATTR-CM: Initial Report From the Open-Label Extension of the ATTRibute-CM Trial
Source: Circulation. 2024 Nov 18;151(9):601–11. doi: 10.1161/CIRCULATIONAHA.124.072771 (PMC11875408; doi:10.1161/CIRCULATIONAHA.124.072771)
Supplement: Supplementary file 1 [file cir-151-601-s001.pdf]

SUPPLEMENTAL MATERIAL

Supplemental Figure 1. Study Design and Eligibility Criteria for Open-Label Extension Trial

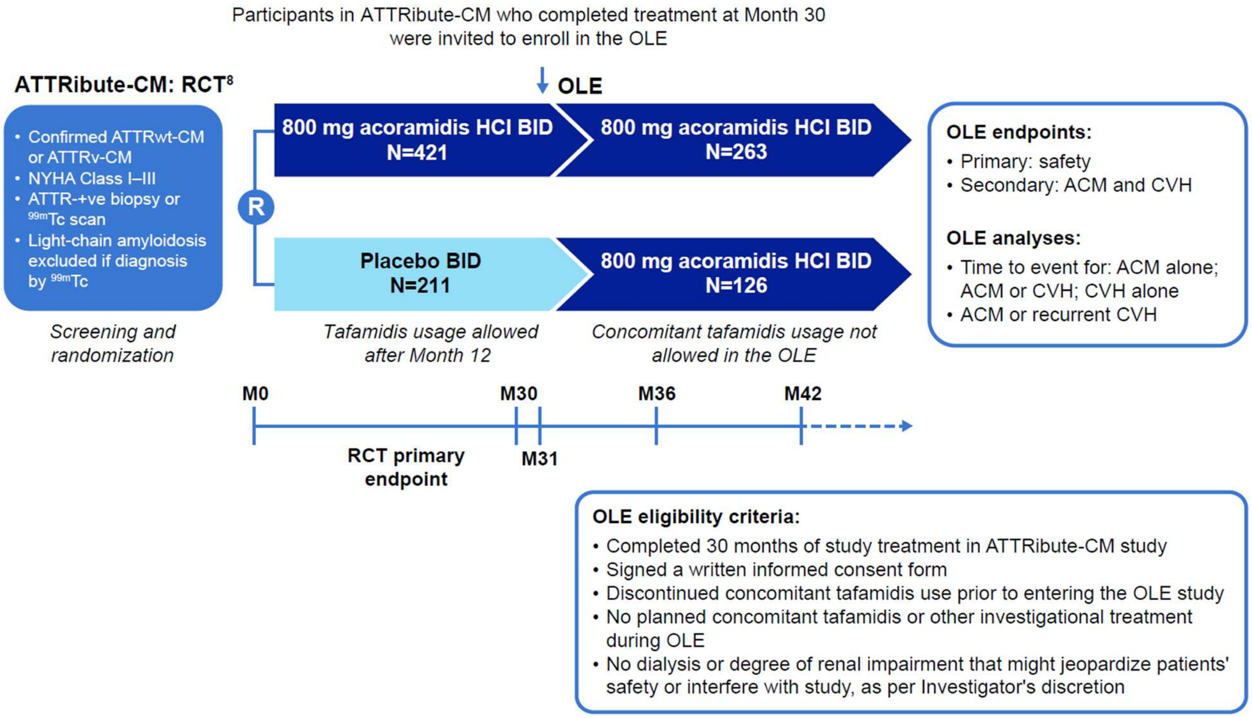

^{99m}Tc, technetium-99m; ACM, all-cause mortality; ATTR, transthyretin amyloidosis cardiomyopathy; ATTRwt-CM, transthyretin amyloidosis wild-type cardiomyopathy; BID, twice daily; CVH, cardiovascular-related hospitalization; HCl, hydrochloric acid; M, month; NYHA, New York Heart Association; OLE, open-label extension; RCT, randomized controlled trial.

Supplemental Figure 2. CONSORT Diagram of Randomization in ATTRibute-CM and Enrollment in the Open-Label Extension Study

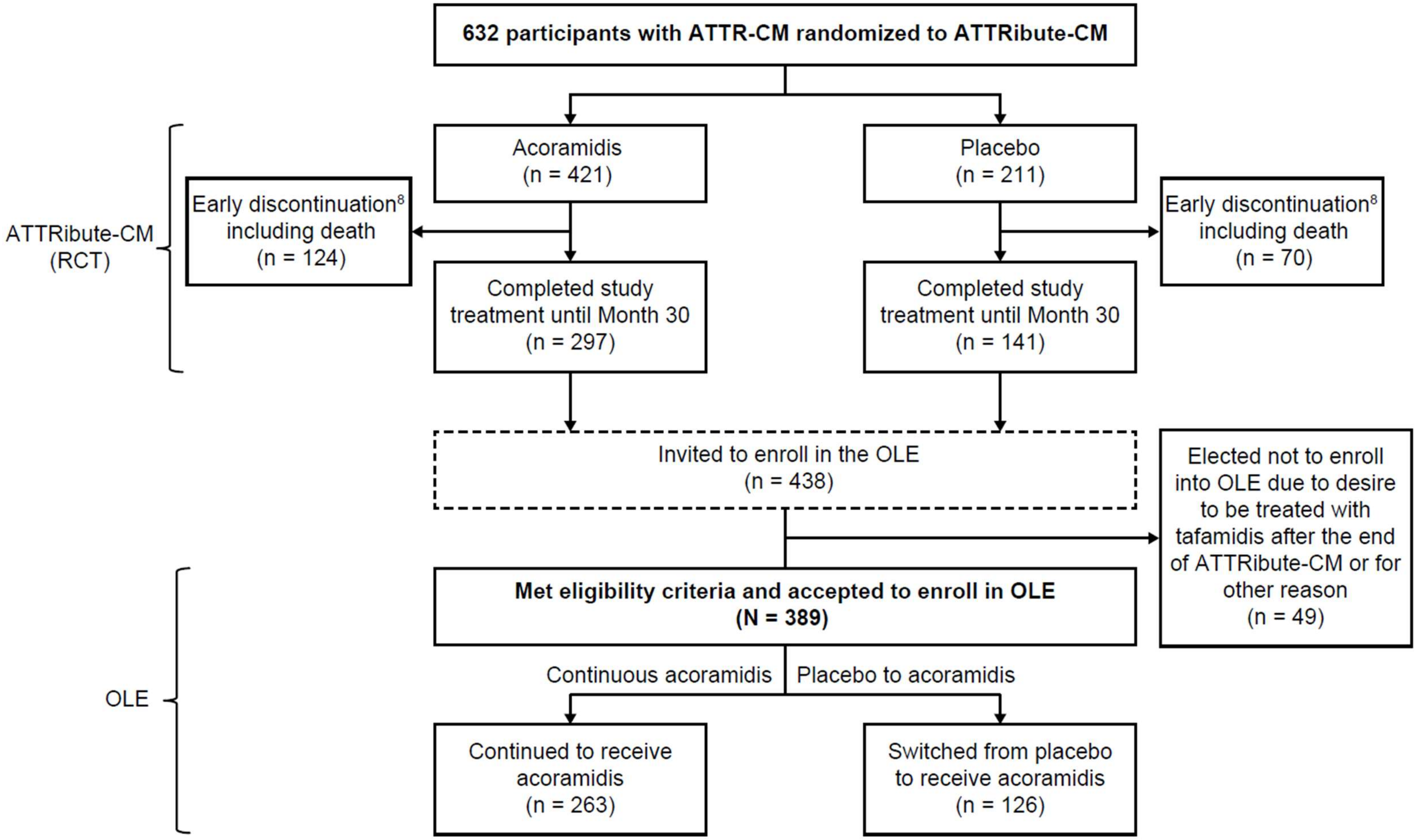

ATTR-CM, transthyretin amyloid cardiomyopathy; OLE, open-label extension; RCT, randomized controlled trial.

Supplemental Table 1. Clinical Outcomes of ACM Alone, ACM or First CVH, and First CVH Alone Through Month 36 and Month 42 in the Open-Label Extension Study

Outcome through M36 in the OLE	Continuous Acoramidis n = 409	Placebo to Acoramidis n = 202
**ACM, n (%)**	82 (20.0)	63 (31.2)
RRR	35.7	
HR (95% CI)*	0.64 (0.46, 0.89)	
**ACM or first CVH, n (%)**	157 (38.4)	118 (58.4)
RRR	34.3	
HR (95% CI)*	0.59 (0.46, 0.75)	
**First CVH, n (%)**	118 (28.9)	98 (48.5)
RRR	40.5	
HR (95% CI)*	0.56 (0.42, 0.73)	
**ACM or Recurrent CVH, n (%)**	157 (38.4)	118 (58.4)
RRR[§]	46%	
**Relative Risk Ratio** (95% CI)[‡]	0.54 (0.40, 0.73)	

Outcome through M42 in the OLE	Continuous Acoramidis n = 409	Placebo to Acoramidis n = 202
**ACM, n (%)**	94[†] (23.0)	70 (34.7)
RRR	33.7	
HR (95% CI)*	0.64 (0.47, 0.88)	
**ACM or first CVH, n (%)**	174[†] (42.5)	130 (64.4)
RRR	33.9	
HR (95% CI)*	0.57 (0.46, 0.72)	
**First CVH, n (%)**	129 (31.5)	108 (53.5)
RRR	41.0	
HR (95% CI)*	0.53 (0.41, 0.69)	
**ACM or Recurrent CVH, n (%)**	174 (42.5)	130 (64.4)
RRR[§]	48%	
**Relative Risk Ratio** (95% CI)[‡]	0.52 (0.39, 0.68)	

*Analyzed using a stratified Cox proportional regression analysis.

[†]One ACM event occurred after M42, but was included in the M42 data cut.

[‡]Analyzed by the negative binomial regression model with treatment group, randomization stratification factors of genotype, NT-proBNP level and eGFR level, and the offset term based on the logarithm of the follow-up duration in years.

[§]RRR is calculated by (1-relative risk ratio from the negative binomial regression analysis) × 100%.

ACM, all-cause mortality; CI, confidence interval; CVH, cardiovascular-related hospitalization; eGFR, estimated glomerular filtration rate; HR, hazard ratio; M, month; NT-proBNP, N-terminal pro-B-type natriuretic peptide; OLE, open-label extension; RRR, relative risk reduction.
